# Head-to-Head Comparison of Consensus-Recommended Platelet Function Tests to Assess P2Y_12_ Inhibition—Insights for Multi-Center Trials

**DOI:** 10.3390/jcm9020332

**Published:** 2020-01-24

**Authors:** Jean-Christophe Bélanger, Fabio Luiz Bandeira Ferreira, Mélanie Welman, Rahma Boulahya, Jean-François Tanguay, Derek Y.F. So, Marie Lordkipanidzé

**Affiliations:** 1 Montreal Heart Institute Research Center, Montréal, QC H1T 1C8, Canada; jean-christophe.belanger@umontreal.ca (J.-C.B.); faluband@gmail.com (F.L.B.F.); melanie.welman@icm-mhi.org (M.W.); rahma.boulahya@umontreal.ca (R.B.); jean-francois.tanguay@icm-mhi.org (J.-F.T.); 2 Faculty of Pharmacy, Université de Montréal, Montréal, QC H3C 3J7, Canada; 3 Institut Armand-Frappier Santé et Biotechnologie-INRS, Laval, QC H7V 1B7, Canada; 4 Faculty of Medicine, Université de Montréal, Montréal, QC H3C 3J7, Canada; 5 Division of Cardiology, University of Ottawa Heart Institute, Ottawa, ON K1Y 4W7, Canada; dso@ottawaheart.ca

**Keywords:** ELISA, flow cytometry, multi-center studies, Multiplate^®^, P2Y12 inhibitors, VASP

## Abstract

The vasodilator-associated stimulated phosphoprotein (VASP) phosphorylation level is a highly specific method to assess P2Y12 receptor inhibition. Traditionally, VASP phosphorylation is analyzed by flow cytometry, which is laborious and restricted to specialized laboratories. Recently, a simple ELISA kit has been commercialized. The primary objective of this study was to compare the performance of VASP assessment by ELISA and flow cytometry in relation to functional platelet aggregation testing by Multiplate^®^ whole-blood aggregometry. Blood from 24 healthy volunteers was incubated with increasing concentration of a P2Y12 receptor inhibitor (AR-C 66096). Platelet function testing was carried out simultaneously by Multiplate^®^ aggregometry and by VASP assessment through ELISA and flow cytometry. As expected, increasing concentrations of the P2Y12 receptor inhibitor induced a proportional inhibition of platelet aggregation and P2Y12 receptor activation across the modalities. Platelet reactivity index values of both ELISA- and flow cytometry-based VASP assessment methods correlated strongly (*r* = 0.87, *p* < 0.0001) and showed minimal bias (1.05%). Correlation with Multiplate^®^ was slightly higher for the flow cytometry-based VASP assay (*r* = 0.79, *p* < 0.0001) than for the ELISA-based assay (*r* = 0.69, *p* < 0.0001). Intraclass correlation (ICC) was moderate for all the assays tested (ICC between 0.62 and 0.84). However, categorization into low, optimal, or high platelet reactivity based on these assays was strongly concordant (κ between 0.86 and 0.92). In conclusion, the consensus-recommended assays with their standardized cut-offs should not be used interchangeably in multi-center clinical studies but, rather, they should be standardized throughout sites.

## 1. Introduction

The 2010 Working Group on High On-Treatment Platelet Reactivity, the 2015 Working Group on Thrombosis of the European Society of Cardiology, and the 2019 Expert Consensus on Platelet Function and Genetic Testing for Guiding P2Y_12_ Receptor Inhibitor Treatment each published position papers on platelet function testing in patients on P2Y_12_ inhibitors. These papers suggested that platelet function could be tailored within a therapeutic window to balance ischemic versus bleeding complications [1,2,3]. Despite recognition that platelet responses to P2Y_12_ inhibitors are highly variable and that high and low platelet reactivity are associated with thrombotic and bleeding events, respectively [4], it is nonetheless challenging to integrate platelet function testing modalities in large multi-center clinical trials assessing different antiplatelet therapies. Expert consensus papers have recommended the use of the VerifyNow^®^ P2Y_12_ assay, the Multiplate^®^ device with the ADP kit, or the vasodilator-stimulated phosphoprotein (VASP) assay, as these have been proven to have the highest association with thrombotic or bleeding outcomes [4,5]. However, previous large randomized trials using the VerifyNow^®^ P2Y_12_ assay to personalize P2Y_12_ therapy have yielded disappointing results [6], thus limiting the clinical utility of VerifyNow^®^. The evidence for the Multiplate^®^ and the VASP assay is sparser but appears promising [4,6].

The traditional VASP assay is amenable to implementation in multi-center trials but requires the shipment of samples to a flow cytometry-ready core facility. Recently, a simple ELISA kit has been commercialized that could allow a wider use of VASP outside of specialized laboratories. The Multiplate^®^ assay can also be carried out locally but it would require more elaborate on-site testing and technical skills. The major advantage of the Multiplate^®^ assay is that it has a functional readout of residual platelet reactivity versus pharmacological P2Y_12_ receptor activity for the VASP assay [7]. We thus sought to investigate whether the results from these assays could be used interchangeably to identify patients with high, optimal, and low platelet reactivity in the context of multi-center studies. Specifically, we compared the performance of VASP assessment by ELISA and flow cytometry, in relation to the functional platelet aggregation testing by Multiplate^®^ whole-blood aggregometry.

## 2. Experimental Section

### 2.1. Study Design

This study was approved by the Research Ethics Committee of the Montreal Heart Institute (#2017-2154, approval date 3 July 2017) and was conducted in accordance with the Declaration of Helsinki. Informed consent was obtained from each participant.

### 2.2. Blood Collection

Twenty-four healthy volunteers were recruited for this study. Venous blood was collected into: 0.109 M, 3.2% sodium citrate (2.7 mL Vacutainer plastic citrate tube, Becton Dickinson, Franklin Lakes, NJ, USA), and hirudin (recombinant hirudin 3.0 mL; Roche Verum Diagnostica, Munich, Germany). Blood samples were incubated with increasing concentrations (0, 0.1, 0.5, 1, 10 µM) of a directly active, potent, and selective P2Y_12_ inhibitor (AR-C 66096 tetrasodium salt; Tocris, Oakville, ON, Canada) for 1 h at room temperature. Immediately after incubation, aliquots were analyzed as described below.

### 2.3. Platelet Function Testing

#### 2.3.1. Analysis of VASP Phosphorylation by ELISA

The VASP phosphorylation state was determined according to the kit instructions for the ELISA test (CY-QUANT VASP/P2Y12, Stago, France). Optical density was read at 450 nm using an optical reader (Infinite F50, Tecan, Morrisville, NC, USA). A platelet reactivity index (PRI) was calculated according to the following formula:

$$\mathrm{PRI}\left(\%\right)\;=\;\frac{{\mathrm{OD}}_{450\mathrm{nm}}\left[\mathrm{PGE}1\right]-{\mathrm{OD}}_{450\mathrm{nm}}\left[\mathrm{PGE}1+\mathrm{ADP}\right]}{{\mathrm{OD}}_{450\mathrm{nm}}\left[\mathrm{PGE}1\right]-{\mathrm{OD}}_{450\mathrm{nm}}\left[\mathrm{Blank}\right]}$$

where OD_450nm_ represents the optical density in the presence of PGE1 alone [PGE1], PGE1 and ADP simultaneously [PGE1 + ADP], or the blank.

#### 2.3.2. Analysis of VASP Phosphorylation by Flow Cytometry

We further used a standardized flow cytometric assay of platelet VASP phosphorylation, as per the manufacturer’s instructions (Platelet VASP; Stago, France). Flow cytometry (FC) analysis was performed using a MACSQuant Analyzer 10 (Miltenyi Biotec, San Diego, CA, USA). A PRI was calculated using corrected mean fluorescence intensities (MFIc) according to the following formula:

$$\mathrm{PRI}\left(\%\right)=\left[\left({\mathrm{MFIc}}_{\mathrm{PGE}1}-{\mathrm{MFIc}}_{\left(\mathrm{PGE}1+\mathrm{ADP}\right)}\right)/{\mathrm{MFIc}}_{\mathrm{PGE}1}\right]\times 100$$

where MFIc represents the corrected mean fluorescence intensity in the presence of PGE1 alone, (PGE1) or PGE1 and ADP simultaneously (PGE1 + ADP).

#### 2.3.3. Multiplate^®^ Aggregometry

Whole-blood aggregation was determined using the Multiplate^®^ analyzer. The ADP test kit (Roche Diagnostics, Diapharma, West Chester, OH, USA) was used as per the manufacturer’s instructions. Platelet aggregation was reported as arbitrary units (U).

#### 2.3.4. Statistical Analysis

Sample size was based on the recommendations from the Clinical and Laboratory Standards Institute [8]. Twenty-four independent qualified reference individuals with 5 concentration–response samples per participant provided 120 samples for analysis, consistent with guideline provisions for verification of reference intervals in clinical laboratories. Concentration–response curves for each method were fitted using a four-parameter logistic model using Prism 8 (Graphpad Software, Inc., La Jolla, CA, USA). The relationship between ELISA, FC, and Multiplate^®^ was assessed by the Pearson correlation coefficient and a linear regression analysis. Continuous variables were reported as median (interquartile range (IQR]). Inter-method reliability comparing ELISA with FC assays were assessed through Bland–Altman 95% limits of agreement and Intraclass Correlation (ICC). Data were categorized based on cut-offs of <19, 19–46, and >46 U for the Multiplate^®^ analyzer and <16, 16–50, and >50% for the VASP assay for low, optimal, and high platelet reactivity [4]. The agreement between VASP by ELISA and FC and Multiplate^®^ assays was assessed with the kappa statistic. A value of *p* < 0.05 was considered significant. Statistical analyses were performed using IBM SPSS Statistics for Macintosh, Version 25.0 (Armonk, NY, USA).

## 3. Results

### 3.1. Inhibition of Platelet P2Y_12_ ADP Receptor Reactivity

**Figure 1:**
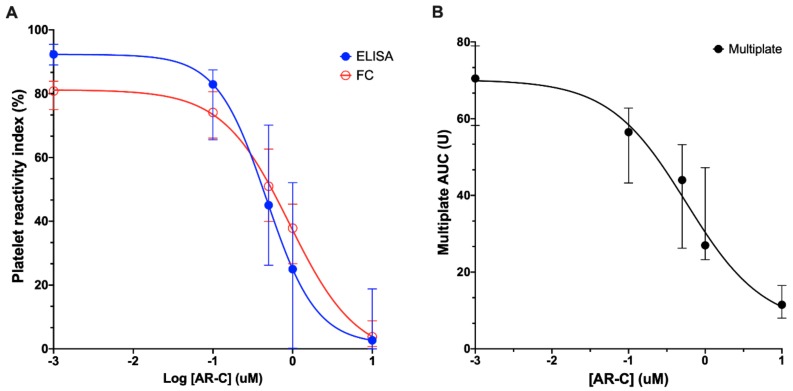
Inhibition of platelet P2Y_12_ activity. (**A**) Concentration–response curves of platelet inhibition by AR-C 66096 as assessed by vasodilator-associated stimulated phosphoprotein (VASP) phosphorylation. Open symbols: flow cytometry. Closed symbols: ELISA. (**B**) Concentration–response curves of platelet inhibition by AR-C 66096 as assessed by the Multiplate^®^ analyzer. Data are presented as median and interquartile range. AUC: area under the curve.

All assays were able to detect inhibition of platelet P2Y_12_ receptors in a concentration-dependent manner (as seen in Figure 1A,B). Incubation with increasing concentrations of AR-C 66096 resulted in a decrease of whole blood aggregation (from 70.5 (58.3–70.0) U to 11.5 (8.0–16.5) U) and PRI (FC: from 81 (75–84)% to 3.8 (0.7–8.8)%; ELISA: from 92 (89–96% to 2.7 (0.0–19)%).

### 3.2. Correlation of FC-VASP with ELISA-VASP

**Figure 2:**
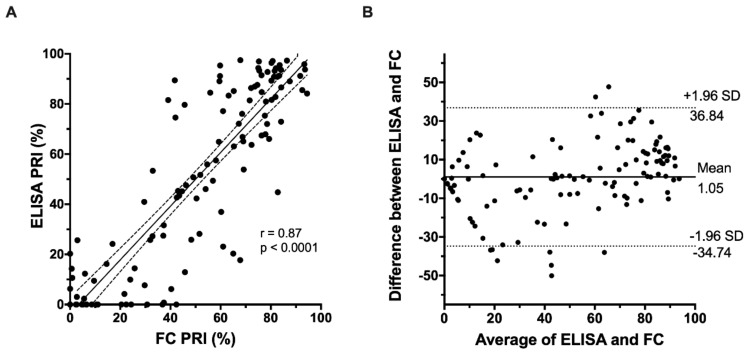
Correlation of FC-VASP with ELISA-VASP (**A**) Linear regression between ELISA-based and flow cytometry-based VASP phosphorylation assessment. (**B**) Bland–Altman assessment of agreement between ELISA-based and flow cytometry-based VASP phosphorylation assessment. PRI: platelet reactivity index.

PRI values measured by ELISA and FC were strongly positively correlated (r = 0.87, *p* < 0.0001, as seen in Figure 2A). Bland–Altman analysis (*n* = 120 observations) was also performed to assess the inter-method reliability of the ELISA and FC assays. The results, as seen in Figure 2B, demonstrate a mean difference between the two assays of 1.05%, with limits of agreement from −34.7% to 36.8%, indicating that systematic bias was minimal, but individual differences could be large.

### 3.3. Correlation of VASP Phosphorylation Assays with the Multiplate^®^ Assay

**Figure 3:**
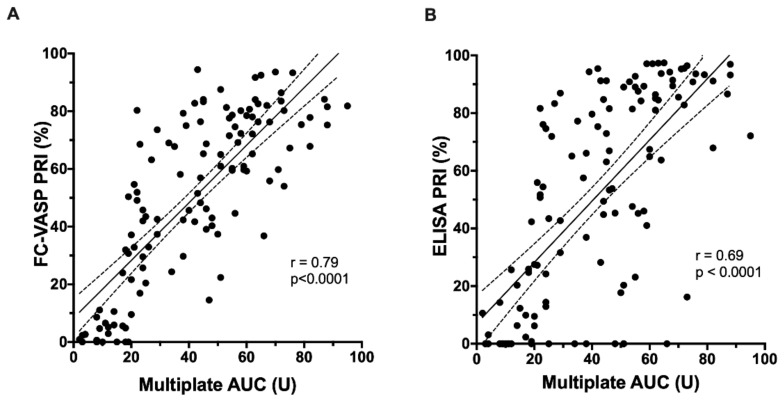
Correlation of VASP phosphorylation assays with the Multiplate^®^ assay. (**A)** Linear regression between flow cytometry-based VASP phosphorylation assessment and Multiplate^®^ analyzer-based aggregation. (**B**) Linear regression between ELISA-based VASP phosphorylation assessment and Multiplate^®^ analyzer-based aggregation.

The correlations between aggregation measured by the Multiplate^®^ analyzer and VASP phosphorylation measured by FC and ELISA were moderately strong (as seen in Figure 3) with FC yielding slightly higher values (FC: *r* = 0.79, *p* < 0.0001; ELISA: *r* = 0.69, *p* < 0.0001).

### 3.4. Classification into High, Optimal, and Low Platelet Reactivity Categories

**Figure 4:**
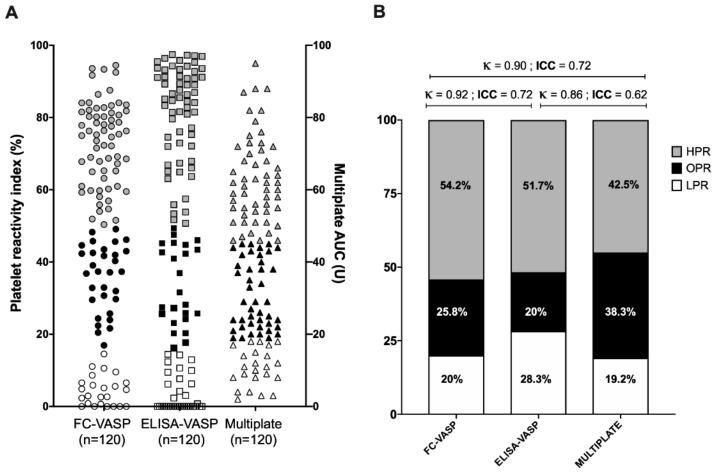
(**A**) Classification of individual samples into high, optimal, and low platelet reactivity. Analysis is based on cut-offs of <19, 19–46, and >46 U for the Multiplate® analyzer and of <16, 16–50, and >50% for the VASP assays for low, optimal, and high platelet reactivity (cut-offs based on Aradi et al. [4]). (**B**) Data expressed as proportion within the therapeutic window by the different platelet function tests.

The platelet function values were divided into categories by using the recommended cut-offs for low, optimal, and high platelet reactivity (as seen in Figure 4A,B) [4]. Between 45% and 54.2% of values fell in the high platelet reactivity category; 20% to 35.8% fell in the optimal range; and 19.2% to 28.3% fell in the low platelet reactivity category. The ICC showed a very high level of reliability of the grouping between ELISA and FC-based VASP methods (ICC = 0.84 (95% CI 0.78 to 0.89); κ = 0.92 (95% CI 0.91 to 0.93)).

Compared to the classification obtained with the Multiplate^®^ analyzer, the similarity with the FC-based VASP classification (ICC = 0.72 (95% CI 0.55 to 0.82); κ = 0.90 (95% CI 0.83 to 0.96)) was moderate and slightly lower with the ELISA-based VASP classification (ICC = 0.62 (95% CI 0.46 to 0.73); κ = 0.86 (95% CI 0.81 to 0.91)).

## 4. Discussion

Platelet function testing has long been confined to specialized laboratories [7]. The need for specialized infrastructure, experienced technical skill, and a time-limited window of action of less than 4 h from blood sampling have arguably rendered large-scale platelet function testing a challenge [9]. Moving away from “gold standard” light transmission aggregometry has liberalized platelet function testing use and sparked the development of standardized platelet function assays—partly or fully automated or simplified sufficiently—to be used in standard laboratories or at the point of care. In a large collective analysis, Aradi et al. pooled together individual data from 17 separate studies, including >20,000 participants [4]. Based on cut-offs of <19, 19–46, and >46 U for the Multiplate^®^ analyzer and of <16, 16–50, and >50% for the VASP assay for low, optimal, and high platelet reactivity, Aradi et al. demonstrated that the risk of stent thrombosis was significantly higher in individuals with high platelet reactivity, with little to no thrombotic benefit of lowering platelet function below the optimal range, whereas bleeding risk was significantly increased [4].

In the context of application in multi-center clinical studies, ease of use, reliability, and accuracy are important considerations when selecting a platelet function assay. Accordingly, we compared the performance of the traditional FC-based VASP assay requiring access to a flow cytometry-capable facility to that of a simplified ELISA-based VASP assay. In terms of practical use, the ELISA assay required significantly less technical skill and dedicated infrastructure than the FC assay. We found their results to be strongly correlated. This conclusion is in agreement with previous studies that also found the assays to be highly concordant [10,11,12]. Indeed, Ding et al. reported a correlation coefficient of 0.89 between the two VASP assays in healthy volunteers, which is very close to our coefficient (0.87). Notwithstanding this fact, once categorized into high, optimal, and low reactivity, we saw an overrepresentation of individuals in the low platelet reactivity category with ELISA-based VASP versus FC-based VASP and Multiplate^®^. An adjustment to cut-off values for ELISA-based VASP may thus be required.

The main drawback of the VASP assay is that it is a pharmacological readout that does not necessarily translate into a pharmacodynamic readout [13]. For this reason, we compared the performance of ELISA- and FC-based VASP assays against the other consensus-recommended whole-blood aggregation assay, i.e., the Multiplate^®^ analyzer. We found the VASP assay to slightly overestimate the number of values in the high and low platelet reactivity categories, with fewer valued in the optimal range. Like Mingant et al., we noted an ICC that varied between 0.62 and 0.84 [13], suggesting that while the agreement is moderate, it is not perfect. 

As recent guidelines do not recommend routine platelet function testing, the current applications of these results would mainly target the research community interested in multi-center studies. We recognize that these results were generated in vitro, which is both a strength and a weakness of our design. While our analysis allowed direct head-to-head comparison of the same samples with different platelet function modalities, it may not capture the same wide array of in vivo platelet responses to P2Y_12_ inhibitors. We have, however, covered the complete range of platelet P2Y_12_ receptor activity from 0% to 100%, and the results obtained extend those of investigators who have studied patients on P2Y_12_ inhibitors [13].

## 5. Conclusions

Both analytical methods of VASP assessment (ELISA and flow cytometry) are reliable, practical, and accurate in determining inhibition of P2Y_12_ receptors, thus allowing for a wider use of VASP outside of specialized laboratories. The choice of one versus the other can therefore be based on the pragmatic grounds of feasibility. The selection of VASP assays as opposed to Multiplate^®^ will require careful concomitant studies with clinical outcomes to assess clinical utility [14]. Therefore, the consensus-recommended assays with their standardized cut-offs should not be used interchangeably in multi-center clinical studies but, rather, they should be standardized throughout sites.
